# Targeting of proteins to the twin‐arginine translocation pathway

**DOI:** 10.1111/mmi.14461

**Published:** 2020-02-20

**Authors:** Tracy Palmer, Phillip J. Stansfeld

**Affiliations:** ^1^ Faculty of Medical Sciences Centre for Bacterial Cell Biology Biosciences Institute, Molecular and Cellular Microbiology Theme Newcastle University Newcastle upon Tyne England; ^2^ School of Life Sciences and Department of Chemistry University of Warwick Coventry UK

**Keywords:** folded protein, mechanism, protein transport, Tat pathway, twin‐arginine signal peptide

## Abstract

The twin‐arginine protein transport (Tat pathway) is found in prokaryotes and plant organelles and transports folded proteins across membranes. Targeting of substrates to the Tat system is mediated by the presence of an N‐terminal signal sequence containing a highly conserved twin‐arginine motif. The Tat machinery comprises membrane proteins from the TatA and TatC families. Assembly of the Tat translocon is dynamic and is triggered by the interaction of a Tat substrate with the Tat receptor complex. This review will summarise recent advances in our understanding of Tat transport, focusing in particular on the roles played by Tat signal peptides in protein targeting and translocation.

## Graphical abstract

The twin‐arginine protein transport (Tat) pathway transports folded proteins across membranes. Targeting of substrates to the Tat system is mediated by the presence of an N‐terminal signal sequence containing a highly conserved twin‐arginine motif. The Tat machinery comprises membrane proteins from the TatA and TatC families. This review will summarise recent advances in our understanding of Tat transport.

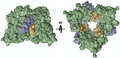

## 1 INTRODUCTION

**Figure 1:**
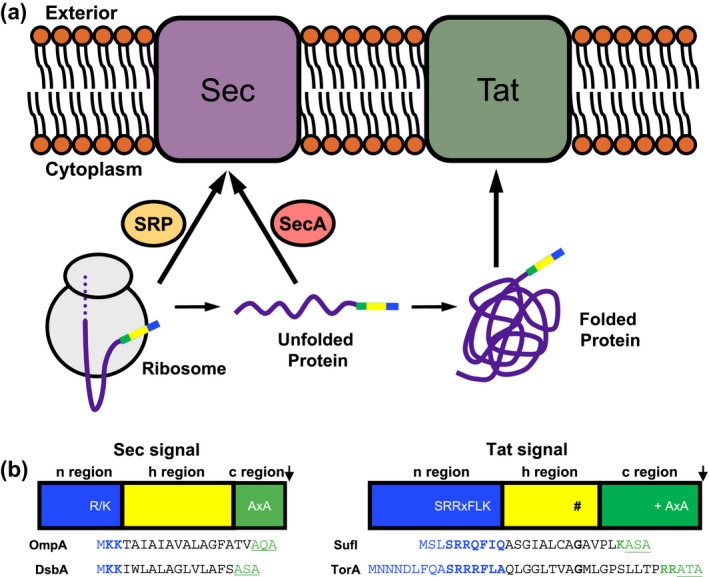
Targeting to the Sec and Tat pathways. (a) The Sec pathway transports unfolded proteins. During co‐translational targeting to Sec, the signal sequence is recognised at the translating ribosome by ribosome‐bound signal recognition particle (SRP) and the nascent chain is guided via the SRP receptor to the Sec translocon, where the energy of protein synthesis is harnessed to drive protein transport. In the post‐translational pathway, the substrate is maintained in an unfolded conformation and guided to the Sec translocon by the ATPase, SecA. ATP hydrolysis by SecA provides the driving force for Sec‐dependent post‐translational protein export (Collinson et al., 2015; Lycklama a Nijeholt & Driessen, 2012; Rapoport, Li, & Park, 2017; Tsirigotaki et al., 2017). The Tat pathway transports folded proteins without the requirement for targeting factors. (b) Signal peptides that target to Sec and Tat pathways share a similar tripartite organisation with a positively charged n‐region, hydrophobic h‐region and polar c‐region containing a signal peptidase cleavage site (AxA). Tat signal peptides have an almost invariant pair of arginines that are embedded within a SRRxFLK motif (Berks, 1996). A helix destabilising residue (#), often a glycine, serine or proline towards the C‐terminal end of the h‐region, provides flexibility at this region of the signal peptide (Hamsanathan et al., 2017). A basic residue (+) is frequently found in the Tat signal peptide c‐region and serves as a Sec avoidance motif (Bogsch et al., 1997). The arrow indicates the position of signal peptide cleavage. Amino acid sequences of two *E. coli* Sec signal peptides, OmpA (post‐translational Sec targeting; Fekkes et al., 1998) and DsbA (co‐translational targeting; Schierle et al., 2003)—basic residues in the n‐region and the signal peptidase cleavage site in the c‐region are underlined and shown in bold. Two well‐studied *E. coli* Tat signal peptides, SufI and TorA, are also shown. Residues that match the twin‐arginine consensus are in red, the Sec avoidance signal in bold typeface and the signal peptidase cleavage site in underline

The transport of proteins across lipid membranes is an essential biological process. In prokaryotes, the general secretory (Sec) and twin‐arginine translocation (Tat) pathways operate in parallel to transport proteins across the cytoplasmic membrane. The Sec system transports unfolded proteins through a narrow channel, in a process that can either be co‐translational or post‐translational (Figure 1a; reviewed in Collinson, Corey, & Allen, 2015; Tsirigotaki, Geyter, Sostaric, Economou, & Karamanou, 2017). Following translocation, proteins fold in the extracellular compartment, usually aided by periplasmic chaperones (Stull, Betton, & Bardwell, 2018). The Tat pathway, by contrast, exports proteins that are folded in the cytosol and is therefore strictly post‐translational (Figure 1a; Berks, 2015; Cline, 2015; Hamsanathan & Musser, 2018). Both of these pathways are also able to integrate hydrophobic segments of transmembrane proteins into the bilayer.

**Figure 2:**
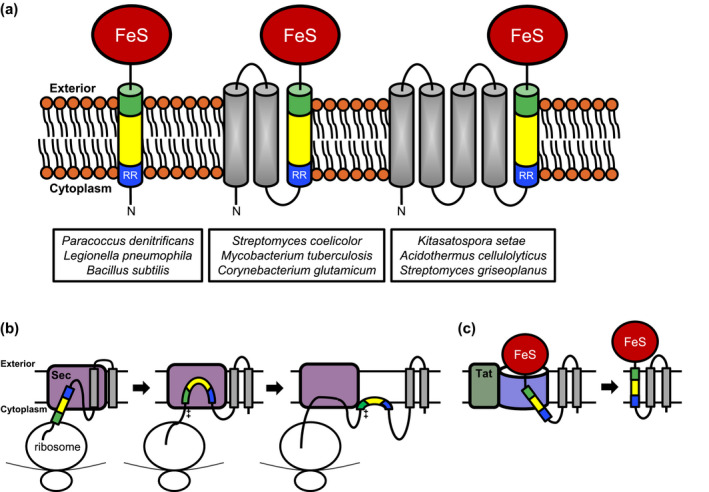
The Sec and Tat pathways cooperate for the biogenesis of some bacterial Rieske proteins. (a) The topological arrangements of selected bacterial Rieske proteins are shown. The vast majority of bacterial Rieske proteins have a single N‐terminal transmembrane helix that is inserted in the membrane by the Tat pathway (Bachmann et al., 2006; De Buck et al., 2007; Goosens, Monteferrante, & Dijl, 2014). Most Actinobacterial Rieske proteins have three transmembrane helices, but some have five (Keller et al., 2012; Tooke et al., 2017). In each case, the final transmembrane helix is integrated by Tat. (b) Biogenesis pathway for polytopic Rieske proteins through Sec, to enable initial transmembrane helix insertion (adapted from Keller et al., 2012; Tooke et al., 2017) and subsequently, (c) through Tat to allow insertion of the final transmembrane helix and transport of the soluble domain

The Sec pathway is ubiquitous and essential as the majority of both extracytoplasmic proteins and polytopic membrane proteins require this system for their localisation. The Tat pathway transports a smaller number of substrates and, as a consequence, is not essential for survival under most growth conditions (reviewed in Palmer & Berks, 2012). Indeed, the Tat pathway is absent from some classes of bacteria and archaea. Nevertheless, the Tat system plays an important role in prokaryote physiology as it transports a subset of proteins that must be exported in a folded state. These proteins are primarily those that noncovalently bind prosthetic groups in the cytoplasm, for example, iron sulphur clusters or the molybdopterin cofactor (Sargent et al., 1998; Weiner et al., 1998). One of the most important Tat substrates is the Rieske iron‐sulphur protein (Figure 2a) of the cytochrome *bc*_1_ and *b*_6_ *f* respiratory complexes which is essential for bacterial photosynthesis and for many types of respiratory metabolism (Aldridge, Spence, Kirkilionis, Frigerio, & Robinson, 2008; Bachmann, Bauer, Zwicker, Ludwig, & Anderka, 2006; De Buck et al., 2007; Hinsley, Stanley, Palmer, & Berks, 2001; Keller, Keyzer, Driessen, & Palmer, 2012; Meloni et al., 2003). The Tat machinery is conserved in the chloroplasts and mitochondria of plants, but with the exception of homoscleromorph sponges it has been lost from the mitochondria of animals (Carrie, Weissenberger, & Soll, 2016; Petru et al., 2018; Pett & Lavrov, 2013; Settles et al., 1997).

## 2 Tat SIGNAL PEPTIDES

Proteins are targeted to the Sec and Tat pathways by N‐terminal signal peptides that are superficially very similar. They each share a tripartite structure with a positively charged n‐region, a hydrophobic h‐region, at least 12 amino acids in length with a propensity for helix formation and a polar c‐region containing a cleavage site for signal peptidase (Dalbey & Wickner, 1985; Lüke, Handford, Palmer, & Sargent, 2009; Yahr & Wickner, 2001; Figure 1b). One of the key differences between the two targeting signals is that the n‐regions of Tat signal peptides almost always have a pair of consecutive arginines, for which, the Tat system is named, whereas the Sec signal peptide n‐regions are simply positively charged with no specific sequence constraints (Berks, 1996). Numerous studies have highlighted the twin‐arginines in Tat signal peptides as essential for efficient Tat transport, with even conservative substitution with lysine being poorly tolerated (e.g., DeLisa, Samuelson, Palmer, & Georgiou, 2002; Stanley, Palmer, & Berks, 2000). By contrast, there appears to be no mechanistic difference between lysine and arginine in Sec signal peptides (Sasaki, Matsuyama, & Mizushima, 1990).

The paired arginines of the Tat signal peptide are part of a larger motif (S‐R‐R‐x‐F‐L‐K; Figure 1b). The amino acids at the other motif positions are only semi‐conserved and none of them are essential for Tat transport (Stanley et al., 2000). After the twin‐arginines, the consensus phenylalanine has the highest frequency (e.g., it is found in two thirds of *Escherichia coli* Tat signals; Palmer, Sargent, & Berks, 2010). Mutational analysis has indicated that amino acid hydrophobicity at this position is an important factor and substitutions that reduce hydrophobicity decrease transport efficiency (Stanley et al., 2000).

A second critical difference between Sec and Tat signal peptides is the degree of h‐region hydrophobicity. Highly hydrophobic Sec signal sequences interact with the signal recognition particle (SRP) when they emerge from the ribosome exit tunnel to engage in co‐translational translocation. By contrast, moderately hydrophobic Sec signals escape SRP and mediate post‐translational translocation through interaction with SecA and other chaperones (Tsirigotaki et al., 2017). Tat signal peptide h‐regions are generally less hydrophobic than Sec signals and contain significantly more glycines and fewer leucines (Cristóbal, Gier, Nielsen, & Heijne, 1999). However, there is an overlap in hydrophobicity scores for naturally occurring Sec and Tat signal peptides and more than half of *E. coli* Tat signals are able to mediate some degree of productive engagement with the Sec pathway if they are fused to a Sec‐compatible reporter protein (Tullman‐Ercek et al., 2007).

It was noted recently that Tat signal peptides contain one or more helix‐destabilising residues (e.g., glycine, proline or serine) located between 12 and 17 residues distal to the twin‐arginine motif (Hamsanathan, Anthonymuthu, Bageshwar, & Musser et al., 2017). Evidence discussed below suggests that these residues are required to allow the signal peptide to undergo conformational changes during interaction with the Tat machinery.

Finally, Tat signal peptides frequently contain at least one basic amino acid in their c‐regions. This is not required for targeting to the Tat system and can be readily substituted for a neutral or even negatively charged amino acid without affecting the rate of Tat transport (Stanley et al., 2000). Instead, it has been shown that the positive charge acts as a Sec‐avoidance motif, reducing functional engagement of the signal peptide with the Sec pathway (Blaudeck, Sprenger, Freudl, & Wiegert, 2001; Bogsch, Brink, & Robinson, 1997; Cristóbal et al., 1999) consistent with earlier studies that found that c‐region basic residues interfere with the function of Sec signal peptides (Geller, Zhu, Cheng, Kuhn, & Dalbey, 1993; Li, Beckwith, & Inouye, 1988).

## 3 FUNCTIONAL OVERLAP BETWEEN Tat AND Sec TARGETING SEQUENCES

In prokaryotes and plastids, the Tat pathway always coexists with Sec. It is imperative that Sec and Tat substrate proteins are sorted to the correct transport pathway. The Sec system cannot tolerate folded proteins, which can lead to lethal jamming of the machinery (Cosma, Danese, Carlson, Silhavy, & Snyder, 1995; van Stelten, Silva, Belin, & Silhavy, 2009), whereas the Tat system is unable to transport most unfolded proteins (e.g., DeLisa, Tullman, & Georgiou, 2003; Halbig, Wiegert, Blaudeck, Freudl, & Sprenger, 1999; Santini et al., 1998). Correct targeting is particularly relevant in the case of an unusual class of cytoplasmic polytopic membrane proteins that rely on the activity of both Sec and Tat for their correct assembly. This class is exemplified by the Rieske iron‐sulphur protein from Actinobacteria. Most Rieske proteins have a single transmembrane domain at their N‐terminus, which in bacteria and plant thylakoids is an uncleaved Tat signal sequence (Bachmann et al., 2006; De Buck et al., 2007; Molik, Karnauchov, Weidlich, Herrmann, & Klösgen, 2001). However, Actinobacterial Rieske proteins, usually have three or occasionally five transmembrane helices preceding the iron sulphur cluster‐containing domain (Figure 2a; Hopkins, Buchanan, & Pamer, 2014; Keller et al., 2012; Niebisch & Bott, 2001; Tooke, Babot, Chandra, Buchanan, & Palmer, 2017). These unusually long Rieske proteins are initially handled like most other polytopic membrane proteins, with the highly hydrophobic first transmembrane helix engaging SRP for co‐translational membrane insertion by the Sec pathway. However, the final transmembrane helix resembles a typical Tat signal sequence, in accord with a requirement for extracellular iron sulphur proteins to acquire their cofactors in the cytoplasm (Berks, 1996; Berks, Sargent, & Palmer, 2000; Keller et al., 2012). Detailed mechanistic analysis has indicated that it is *a combination* of low relative hydrophobicity coupled with the presence of numerous positive charges at the C‐terminal side of the helix that render the Sec system unable to fully translocate the C‐terminus of this domain across the membrane. As a result, the Sec apparatus releases the transmembrane domain which most probably forms a re‐entrant loop in the membrane (Tooke et al., 2017; Figure 2b). Following cofactor insertion into the Rieske domain, the membrane‐tethered Tat signal sequence is recognised by the Tat system to complete localisation and assembly of the protein (Figure 2b).

The finding that the Tat signal peptides of such dual‐targeted proteins initially engage with the Sec apparatus has significance for the targeting mechanism of soluble Tat substrates. Analysis of the known and predicted Tat substrates in *E. coli* and *Salmonella* shows that the overwhelming majority have the basic c‐region or mature domain N‐terminus required to avoid Sec transport. The inference is that such signal peptides often initially engage with the Sec pathway and abort at a late stage when the C‐terminal positive charges are recognised and are inserted in the membrane as re‐entrant loops. This would mean that the Tat system frequently recognises membrane‐associated signal peptides (Bageshwar, Whitaker, Liang, & Musser, 2009; Ma & Cline, 2000; Musser & Theg, 2000; Shanmugham, Wong Fong Sang, Bollen, & Lill, 2006).

Despite the overwhelming conservation of the twin‐arginines in Tat signal peptides, recent studies demonstrate that they are not mechanistically essential for operation of the Tat pathway. Specifically, inactivating substitutions in either the paired arginines or their binding site in the Tat translocon can be overcome by increasing the hydrophobicity of the signal peptide h‐region (Huang & Palmer, 2017; Ulfig et al., 2017). Some of these hydrophobic suppressors are able to direct significant levels of export, approaching 30% of wild type transport activity and it was noted that even signal peptides with a marked increase in hydrophobicity (approaching those that target the SRP pathway) could productively engage with Tat (Huang & Palmer, 2017; Ulfig et al., 2017).

Collectively these results indicate that the functional requirements for Tat signal peptides are remarkably similar to Sec, that is, one or more positive charge in the signal peptide n‐region coupled with a relatively hydrophobic h‐region. Indeed, it has been shown that two canonical Sec signal peptides, OmpA and DsbA (Figure 1b) are able to interact with the Tat machinery and mediate Tat‐dependent transport of a reporter protein. A conservative estimate predicts that almost half of *E. coli* Sec signals have features that would permit engagement with the Tat pathway (Huang & Palmer, 2017). However, in vivo, it is unlikely that such substrates would ever reach the Tat machinery because their signal peptides would interact with either SRP or SecA and be channelled into the Sec pathway. This places extraordinary constraints on Tat signal sequences which must evolve to escape recognition by these targeting factors. Indeed, it is likely that the twin‐arginine motif and its cognate recognition site on the translocon, arose to increase the affinity of the Tat system for the weakly hydrophobic signal peptides.

By the same token, although signal peptides with paired arginines are compatible with the Sec pathway, only 0.02% of *E. coli* Sec signals contain this feature, whereas paired lysines are much more common (Huang & Palmer, 2017). This implies that there may also be evolutionary constraints acting on Sec targeting sequences. Interestingly, while some Tat substrate proteins are clearly incompatible with the Sec pathway because they must be folded in the cytoplasm, some protein families are compatible with either export route. A good example of this is the cell wall amidase family which in *E. coli* has the three members; AmiA, AmiB and AmiC. While AmiA and AmiC are Tat substrates, AmiB (which has 40% sequence identity to AmiC), is a Sec substrate (Bernhardt & de Boer, 2003; Ize, Stanley, Buchanan, & Palmer, 2003). Similarly, many solute binding proteins that would normally be expected to utilise the Sec pathway are Tat substrates in *Streptomyces* bacteria. (Joshi et al., 2010; Widdick et al., 2006). Intriguingly, the Tat machinery is localised to the tips of growing hyphae in *Streptomyces coelicolor*, so it is plausible that the Tat‐dependent export of these proteins may reflect a requirement for them to be secreted at the region of active growth (Willemse et al., 2012).

## 4 Tat SIGNAL PEPTIDES TRIGGER ASSEMBLY OF THE ACTIVE Tat TRANSLOCON

**Figure 3:**
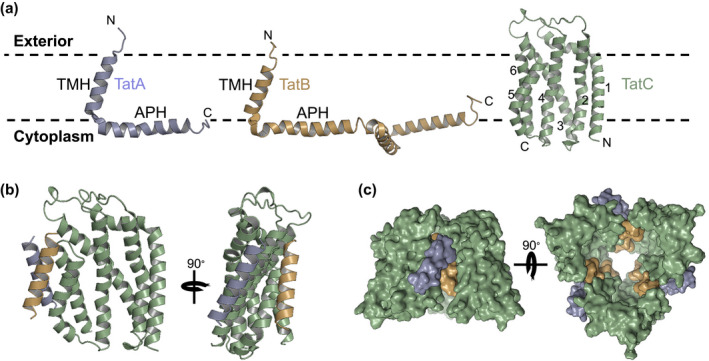
Structures of the Tat components and a model for the Tat receptor complex. (a) Structures of TatA (blue), TatB (orange) and TatC (green) (Ramasamy et al., 2013; Rodriguez et al., 2013; Rollauer et al., 2012; Zhang et al., 2014). TatC has six transmembrane helices with the N‐ and C‐terminus located at the cytoplasmic side of the membrane. Transmembrane helices are numbered. (b) A model for the resting state of the TatABC receptor, showing the interactions of the transmembrane helices, with the constituent subunits coloured as in part A. (c) Location of the TatA and TatB transmembrane helix binding sites on TatC in the resting state receptor. The TatC stoichiometry of the Tat complex is not established, but modelled here for a TatC trimer (adapted from Habersetzer et al., 2017)

The Tat machinery comprises membrane proteins from the TatA and TatC families. TatA proteins are monotopic, with an amphipathic C‐terminal domain located at the cytoplasmic side of the membrane (Aldridge, Storm, Cline, & Dabney‐Smith, 2012; Koch, Fritsch, Buchanan, & Palmer, 2012; Figure 3a). In most Gram‐negative bacteria and some Gram‐positive bacteria (e.g., *Streptomyces*), two functionally distinct TatA family proteins, TatA and TatB, are present (Chanal, Santini, & Wu, 1998; De Keersmaeker et al., 2005; Hicks et al., 2006; Sargent, Stanley, Berks, & Palmer, 1999). By contrast, TatB proteins are not found in the Tat systems of archaea, Gram‐positive bacteria of the firmicutes phylum and some obligate intracellular Gram‐negative bacteria, (Dilks, Gimenez, & Pohlschröder, 2005; Jongbloed et al., 2004; Nunez, Soria, & Farber, 2012). NMR structures of the helical regions of TatA and TatB have been determined (Figure 3a), with both proteins having a relatively short N‐terminal transmembrane helix that is only marginally long enough to span the bilayer (Hu, Zhao, Li, Xia, & Jin, 2010; Rodriguez et al., 2013; Zhang, Wang, Hu, & Jin, 2014).

TatC family proteins are polytopic, with six transmembrane helices. X‐ray structures of *Aquifex aeolicus* TatC show that the six helices form a glove‐shape with the arrangement stabilised by a structured periplasmic cap (Ramasamy, Abrol, Suloway, Clemons, & JR., 2013; Rollauer et al., 2012; Figure 3a). Notably, the final two transmembrane helices of TatC (TM 5 and 6) are of similar length to the N‐terminal transmembrane helices of TatA and TatB and molecular dynamics simulations indicate that the bilayer will be thinned in their vicinity (Ramasamy et al., 2013; Rollauer et al., 2012).

**Figure 4:**
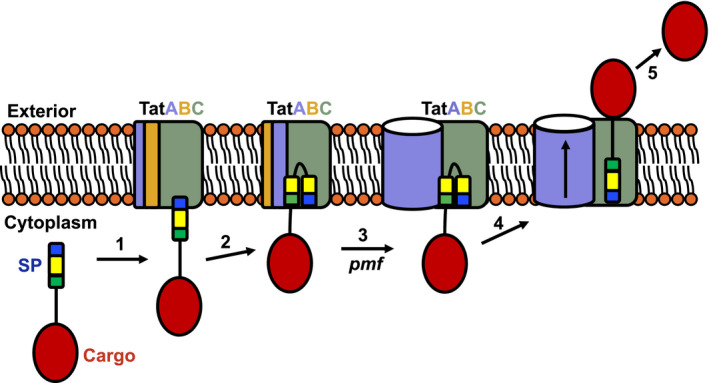
A Model for the Tat transport pathway. Step1. A folded Tat substrate docks at the Tat receptor complex, the twin‐arginines in the signal peptide n‐region binding to the cytoplasmic surface of TatC. Step 2. The signal peptide transitions to bind more deeply into the receptor, inserting in a hairpin conformation. The deep insertion of the signal peptide displaces TatB from its resting state binding site on TatC to occupy the TatA binding site at TMH6. A TatA molecule is now recruited to the binding site vacated by TatB. Step 3. The positioning of TatA at the TM5 binding site allows the further recruitment and nucleation of TatA molecules to form a large oligomer. Step 4. The signal peptide hairpin unhinges and the substrate passes across the membrane facilitated by the TatA oligomer. Step 5. The signal peptide is cleaved and the mature domain is released at the periplasmic side of the membrane. Following substrate translocation, the TatA oligomer dissociates and the Tat receptor returns to the resting state

Most mechanistic studies on the bacterial Tat pathway have used *E. coli* as a model, with plant thylakoids also providing an excellent system for mechanistic analysis of the related eukaryotic Tat pathway. Current evidence points to a model whereby the active Tat translocon assembles ‘on demand’ upon interaction with a substrate protein and that it disassembles once translocation is complete (Alami et al., 2003; Alcock et al., 2013; Mori & Cline, 2002; Rose, Fröbel, Graumann, & Müller, 2013; Figure 4).

In the resting state the Tat receptor complex comprises multiple (probably three or four) copies of TatA, TatB and TatC in a 1:1:1 ratio (Alcock et al., 2016 Bolhuis, Mathers, Thomas, Barrett, & Robinson, 2001; Habersetzer et al., 2017). Crosslinking studies alongside sequence co‐evolution analysis and molecular simulations have generated a robust model for the receptor complex (Alcock et al., 2016; Blümmel, Haag, Eimer, Müller, & Fröbel, 2015, Habersetzer et al., 2017; Figure 3b). In the resting state, the transmembrane domain of TatB makes extensive contacts along the length of transmembrane helix 5 of TatC and the top of helix 6. Contacts between the opposite face of the TatB transmembrane helix and the first transmembrane helix of an adjacent TatC facilitate oligomerisation of the complex (Alcock et al., 2016; Blümmel et al., 2015). While a complex of TatB and TatC is stable to purification and retains the ability to interact with Tat signal peptides (Bolhuis et al., 2001; de Leeuw et al., 2002; Tarry et al., 2009), in vivo TatA is also associated with the complex in the resting state (Alcock et al., 2016; Aldridge, Ma, Gérard, & Cline, 2014; Habersetzer et al., 2017; Zoufaly et al., 2012). The TatA binding site was localised through cysteine crosslinking and molecular modelling to transmembrane helix 6 of TatC, adjacent to the TatB binding site which lies primarily on helix 5 (Figure 3b; Habersetzer et al., 2017).

Tat transport is initiated by the interaction of a Tat signal peptide with the receptor, with the twin‐arginine motif recognised by a conserved surface patch on the cytoplasmic face of TatC (Alami et al., 2003; Rollauer et al., 2012). Following initial binding, the signal peptide subsequently transitions to bind more deeply within the receptor complex (Alami et al., 2003; Blümmel et al., 2015; Gérard & Cline, 2007; Hamsanathan et al., 2017; Figure 4). This promotes a reorganisation of the receptor complex in which TatB is displaced from its resting state binding site on TatC, allowing this site to be occupied by TatA (Alcock et al., 2016; Habersetzer et al., 2017). In this activated state of the receptor complex, we expect that TatB now occupies the helix 6 binding site. Crosslinking experiments also suggest that TatC molecules adopt a tail‐to‐tail orientation following activation, homodimerising through transmembrane helices 5 and 6 (Cléon et al., 2015; Habersetzer et al., 2017; Huang et al., 2017). The precise order of these events is unclear. However, based on current evidence, it is likely that receptor re‐organisation is triggered by interaction of the signal peptide h‐region with TatB, consistent with the extensive contacts TatB makes with this region of the signal peptide (Alami et al., 2003; Gérard & Cline, 2006; Panahandeh, Maurer, Moser, Delisa, & Müller, 2008). This mechanistic model is supported by genetic suppressor analysis, where a group of suppressor substitutions were identified in the transmembrane helix of TatB that restored Tat transport activity to signal peptides with inactivating substitutions of the twin‐arginine motif and to TatC variants that had inactivating substitutions in the twin‐arginine recognition site. Biochemical analysis of these suppressors revealed signal peptide‐independent structural reorganisation of the receptor complex (Huang et al., 2017) and for the strongest suppressor, TatB F13Y, constitutive TatB vacation of the TM5 site and occupancy of the TM6 site (Tooke and Palmer, unpublished).

**Figure 5:**
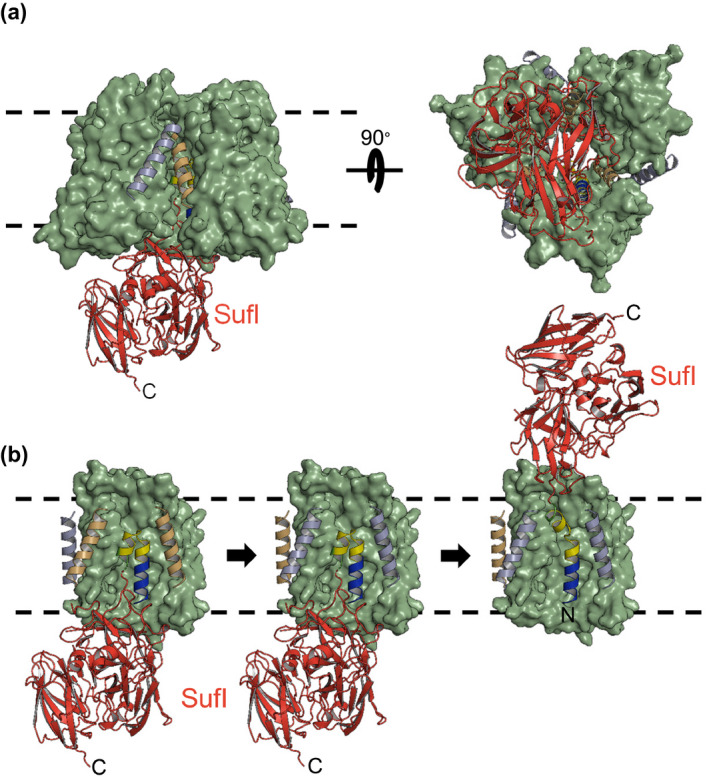
Hypothetical Structural Model for the signal peptide bound receptor complex. (a) Model of the TatABC (blue, orange and green) resting‐state complex, with bound SufI substrate (red). Views are from the membrane (left) and the cytoplasm (right). (b) The signal peptide hairpin of SufI (blue/yellow/green) binds to the concave face of TatC, such that it can interact with both copies of TatB, either side of a TatC monomer (left). The binding of signal peptide induces an exchange between TatA and TatB subunits (middle), with the TatA subunits ultimately oligomerising (not shown) to permit the translocation of the SufI mature domain, aided by the unhinging of the signal peptide hairpin (right)

The structure of the signal peptide‐activated form of the receptor complex is not known. However, it has been shown that covalently attaching the twin‐arginine motif to its binding site on TatC does not inhibit translocation of a substrate across the membrane, indicating that the twin‐arginine residues remain at the cytoplasmic face of the membrane (Gérard & Cline, 2006). Extensive crosslinks have been detected throughout the signal peptide h‐region with TatB (Alami et al., 2003; Gérard & Cline, 2006; Panahandeh et al., 2008) and a site‐specific crosslink observed between a cysteine residue in the C‐terminal end of the h‐region and a cysteine in TatC TM5 (Aldridge et al., 2014). Applying these constraints to modelling the TatC‐signal peptide complex places the signal peptide bound at the cytoplasmic face of one TatC protomer with the h‐region interacting with TatB bound to TM5 of the adjacent TatC protomer (Alcock et al., 2016; Aldridge et al., 2014; Figure 5). The identification of TatC variants that have dominant negative activity (i.e., that inhibit Tat transport activity in the presence of a wild type copy of TatC) strongly supports the inference that hetero‐oligomerisation of the receptor complex is a functional requirement (Cléon et al., 2015).

A recent study has confirmed that Tat signal peptides bind to the receptor complex in a hairpin conformation. Fluorescence quenching experiments place the C‐terminal end of the signal peptide h‐region at the tip of the hairpin, directly preceding the helix‐destabilising residue (Figure 1b; Hamsanathan et al., 2017). The second arm of the hairpin would then be formed from the signal peptide c‐region and potentially residues at the N‐terminus of the mature domain (Figure 5), Indeed, crosslinking of both of these regions to TatB have been detected (Gérard & Cline, 2006; Hamsanathan et al., 2017). A role for the early mature domain of the substrate protein in receptor binding is supported by the isolation of suppressor substitutions in this region that can compensate for inactivating twin‐arginine substitutions (Ulfig & Freudl, 2018). Collectively, these results point to a model, where the signal peptide may make contact with two separate TatB molecules; the c‐region and early mature domain contacting TatB bound to TM5 of the same TatC protomer and the h‐region with TatB bound at TM5 of a neighbouring TatC (Figure 5). In principle, contacts of the signal peptide with one or other of these TatB molecules (or potentially both) could drive productive reorganisation of the receptor.

This signal peptide‐induced structural rearrangement primes the receptor for the recruitment of further TatA molecules, in a process that is dependent on the protonmotive force (Alami et al., 2003; Alcock et al., 2013; Aldridge et al., 2014; Dabney‐Smith & Cline, 2009; Dabney‐Smith, Mori, & Cline, 2006; Mori & Cline, 2002; Rose et al., 2013; Figure 2b). The mechanism of TatA oligomer assembly is not understood. However, it has been speculated that the concave face of TatC may form a platform to support multimerisation (Rollauer et al., 2012). Intriguingly, activation of the receptor complex results in TatA occupancy at the TatC TM5 binding site which lies at the edge of this face and could potentially act as a nucleation point for TatA polymerisation (Alcock et al., 2016; Habersetzer et al., 2017). Some support for this mechanistic model comes from the work of Aldridge et al. who observed recruitment of Tha4 (the thylakoid orthologue of TatA) to a site at the concave face of TatC under protein transport conditions (Aldridge et al., 2014). At present, it is not clear whether TatA forms an oligomer of fixed size or a series of size‐variable assemblies (Beck et al., 2013; Dabney‐Smith et al., 2006; Dabney‐Smith & Cline, 2009; Gohlke et al., 2005; Leake et al., 2008; Oates et al., 2005; Richter & Brüser, 2005). Further studies are required to understand the formation and arrangement of the TatA oligomer.

Evidence suggests that unhinging of the signal peptide hairpin may be a critical step in substrate translocation and deliberate locking of the hairpin by internal crosslinking inhibits transport (Hamsanathan et al., 2017). How transport of the passenger domain is achieved, however, is still open to debate. Mechanisms for substrate transport are discussed in recent reviews (Berks, 2015; Cline, 2015; Hamsanathan & Musser, 2018) and will not be described in detail here. However, according to current models, the assembled TatA oligomer forms the substrate translocation pathway either through formation of a (size‐variable) channel or by promoting localised membrane weakening and transient bilayer disruption (Brüser & Sanders, 2003; Gohlke et al., 2005; Leake et al., 2008; Rodriguez et al., 2013). Following passage of substrate across the membrane, the signal peptide is cleaved (Figure 4), the TatA oligomeric pore dissociates as the assembled translocation system rearranges to the resting state. Currently almost nothing is known about the mechanism of Tat translocon disassembly and whether it is an obligate step for each round of substrate transport.

## 5 FUTURE PERSPECTIVES

Significant progress towards understanding the mechanism of protein transport by the Tat pathway has been catalysed by the determination of high‐resolution structures for TatC and the helical regions of TatA and TatB (Ramasamy et al., 2013; Rodriguez et al., 2013; Rollauer et al., 2012; Zhang et al., 2014). However, currently, we still lack a molecular level understanding of protein translocation, which ideally requires structural resolution of protein complexes and transport intermediates. The highly dynamic nature of the Tat system makes this particularly challenging, but may be facilitated through isolation of mutations that lock the translocon in intermediate states. The identification of a substitution that promotes constitutive translocon assembly (e.g., *E. coli* TatBF13Y; Huang et al., 2017) could offer insight into the nature of the assembled translocon and has the potential to address questions including whether the assembled TatA oligomer is of fixed size and how TatA molecules are scaffolded. Finally, it is unclear how translocon disassembly is initiated and whether this process is related to the mechanism by which the Tat system fails to transport some unfolded proteins (Panahandeh et al., 2008; Richter & Brüser, 2005).

## References

[mmi14461-bib-0001] Alami, M. , Lüke, I. , Deithermann, S. , Eisner, G. , Koch, H. G. , Brunner, J. , & Müller, M. (2003). Differential interactions between a twin‐arginine signal peptide and its translocon in *Escherichia coli* . Molecular Cell, 12, 937–946. 14580344 10.1016/s1097-2765(03)00398-8

[mmi14461-bib-0002] Alcock, F. , Baker, M. A. , Greene, N. P. , Palmer, T. , Wallace, M. I. , & Berks, B. C. (2013). Live cell imaging shows reversible assembly of the TatA component of the twin‐arginine protein transport system. Proceedings of the National Academy of Sciences of the United States of America, 110, E3650–E3659. 10.1073/pnas.1306738110 24003141 PMC3780885

[mmi14461-bib-0003] Alcock, F. , Stansfeld, P. J. , Basit, H. , Habersetzer, J. , Baker, M. A. , Palmer, T. , … Berks, B. C . (2016). Assembling the Tat protein translocon. Elife, 5, pii: e20718. 10.7554/eLife.20718 PMC5201420 27914200

[mmi14461-bib-0004] Aldridge, C. , Ma, X. , Gérard, F. , & Cline, K. (2014). Substrate‐gated docking of pore subunit Tha4 in the TatC cavity initiates Tat translocon assembly. Journal of Cell Biology, 205, 51–65. 24711501 10.1083/jcb.201311057 PMC3987133

[mmi14461-bib-0005] Aldridge, C. , Spence, E. , Kirkilionis, M. A. , Frigerio, L. , & Robinson, C. (2008). Tat‐dependent targeting of Rieske iron‐sulphur proteins to both the plasma and thylakoid membranes in the cyanobacterium *Synechocystis PCC6803* . Molecular Microbiology, 70, 140–150. 10.1111/j.1365-2958.2008.06401.x 18699865

[mmi14461-bib-0006] Aldridge, C. , Storm, A. , Cline, K. , & Dabney‐Smith, C. (2012). The chloroplast twin arginine transport (Tat) component, Tha4, undergoes conformational changes leading to Tat protein transport. Journal of Biological Chemistry, 287, 34752–34763. 10.1074/jbc.M112.385666 22896708 PMC3464578

[mmi14461-bib-0007] Bachmann, J. , Bauer, B. , Zwicker, K. , Ludwig, B. , & Anderka, O. (2006). The Rieske protein from *Paracoccus denitrificans* is inserted into the cytoplasmic membrane by the twin‐arginine translocon. The Febs Journal, 273, 4817–4830. 16987314 10.1111/j.1742-4658.2006.05480.x

[mmi14461-bib-0008] Bageshwar, U. K. , Whitaker, N. , Liang, F. C. , & Musser, S. M. (2009). Interconvertibility of lipid‐ and translocon‐bound forms of the bacterial Tat precursor pre‐SufI. Molecular Microbiology, 74, 209–226. 10.1111/j.1365-2958.2009.06862.x 19732346 PMC2770089

[mmi14461-bib-0009] Beck, D. , Vasisht, N. , Baglieri, J. , Monteferrante, C. G. , van Dijl, J. M. , Robinson, C. , & Smith, C. J. (2013). Ultrastructural characterisation of *Bacillus subtilis* TatA complexes suggests they are too small to form homooligomeric translocation pores. Biochimica et Biophysica Acta, 1833, 1811–1819. 10.1016/j.bbamcr.2013.03.028 23567937 PMC3988878

[mmi14461-bib-0010] Berks, B. C. (1996). A common export pathway for proteins binding complex redox cofactors? Molecular Microbiology, 22, 393–404. 8939424 10.1046/j.1365-2958.1996.00114.x

[mmi14461-bib-0011] Berks, B. C. (2015). The twin‐arginine protein translocation pathway. Annual Review of Biochemistry, 84, 843–864. 10.1146/annurev-biochem-060614-034251 25494301

[mmi14461-bib-0012] Berks, B. C. , Sargent, F. , & Palmer, T. (2000). The Tat protein export pathway. Molecular Microbiology, 35, 260–274. 10.1046/j.1365-2958.2000.01719.x 10652088

[mmi14461-bib-0013] Bernhardt, T. G. , & de Boer, P. A. (2003). The *Escherichia coli* amidase AmiC is a periplasmic septal ring component exported via the twin‐arginine transport pathway. Molecular Microbiology, 48, 1171–1182. 10.1046/j.1365-2958.2003.03511.x 12787347 PMC4428285

[mmi14461-bib-0014] Blaudeck, N. , Sprenger, G. A. , Freudl, R. , & Wiegert, T. (2001). Specificity of signal peptide recognition in tat‐dependent bacterial protein translocation. Journal of Bacteriology, 183, 604–610. 10.1128/JB.183.2.604-610.2001 11133954 PMC94916

[mmi14461-bib-0015] Blümmel, A. S. , Haag, L. A. , Eimer, E. , Müller, M. , & Fröbel, J. (2015). Initial assembly steps of a translocon for folded proteins. Nature Communications, 6, 7234. 10.1038/ncomms8234 PMC4490388 26068441

[mmi14461-bib-0016] Bogsch, E. , Brink, S. , & Robinson, C. (1997). Pathway specificity for a delta pH‐dependent precursor thylakoid lumen protein is governed by a ‘Sec‐avoidance’ motif in the transfer peptide and a ‘Sec‐incompatible’ mature protein. EMBO Journal, 16, 3851–3859. 9233795 10.1093/emboj/16.13.3851 PMC1170009

[mmi14461-bib-0017] Bolhuis, A. , Mathers, J. E. , Thomas, J. D. , Barrett, C. M. , & Robinson, C. (2001). TatB and TatC form a functional and structural unit of the twin‐arginine translocon from *Escherichia coli* . Journal of Bioogicall Chemistry, 276, 20213–20219. 10.1074/jbc.M100682200 11279240

[mmi14461-bib-0018] Brüser, T. , & Sanders, C. (2003). An alternative model of the twin arginine translocation system. Microbiological Research, 158, 7–17. 10.1078/0944-5013-00176 12608575

[mmi14461-bib-0019] Carrie, C. , Weissenberger, S. , & Soll, J. (2016). Plant mitochondria contain the protein translocon subunits TatB and TatC. Journal of Cell Science, 129, 3935–3947. 27609835 10.1242/jcs.190975

[mmi14461-bib-0020] Chanal, A. , Santini, C. , & Wu, L. (1998). Potential receptor function of three homologous components, TatA, TatB and TatE, of the twin‐arginine signal sequence‐dependent metalloenzyme translocation pathway in *Escherichia coli* . Molecular Microbiology, 30, 674–676. 10.1046/j.1365-2958.1998.01095.x 9822832

[mmi14461-bib-0021] Cléon, F. , Habersetzer, J. , Alcock, F. , Kneuper, H. , Stansfeld, P. J. , Basir, H. … Palmer, T . (2015). The TatC component of the twin‐arginine protein translocon functions as an obligate oligomer. Molecular Microbiology, 98, 111–129. 26112072 10.1111/mmi.13106 PMC5102672

[mmi14461-bib-0022] Cline, K. (2015). Mechanistic aspects of folded protein transport by the twin arginine translocon (Tat). Journal of Biological Chemistry, 290, 16530–16538. 25975269 10.1074/jbc.R114.626820 PMC4505407

[mmi14461-bib-0023] Collinson, I. , Corey, R. A. , & Allen, W. J. (2015). Channel crossing: How are proteins shipped across the bacterial plasma membrane? Philosphical Transactions of the Royal Society of London Series B Biological Sciences, 370, pii: 20150025 10.1098/rstb.2015.0025 PMC4632601 26370937

[mmi14461-bib-0024] Cosma, C. L. , Danese, P. N. , Carlson, J. H. , Silhavy, T. J. , & Snyder, W. B. (1995). Mutational activation of the Cpx signal transduction pathway of *Escherichia coli* suppresses the toxicity conferred by certain envelope‐associated stresses. Molecular Microbiology, 18, 491–505. 10.1111/j.1365-2958.1995.mmi_18030491.x 8748033

[mmi14461-bib-0025] Cristóbal, S. , de Gier, J. W. , Nielsen, H. , & von Heijne, G. (1999). Competition between Sec‐ and TAT‐dependent protein translocation in *Escherichia coli* . EMBO Journal, 18, 2982–2990. 10.1093/emboj/18.11.2982 10357811 PMC1171380

[mmi14461-bib-0026] Dabney‐Smith, C. , & Cline, K. (2009). Clustering of C‐terminal stromal domains of Tha4 homo‐oligomers during translocation by the Tat protein transport system. Molecular Biology of the Cell, 20, 2060–2069. 10.1091/mbc.e08-12-1189 19193764 PMC2663938

[mmi14461-bib-0027] Dabney‐Smith, C. , Mori, H. , & Cline, K. (2006). Oligomers of Tha4 organize at the thylakoid Tat translocon during protein transport. Journal of Biological Chemistry, 281, 5476–5483. 16407186 10.1074/jbc.M512453200

[mmi14461-bib-0028] Dalbey, R. E. , & Wickner, W. (1985). Leader peptidase catalyzes the release of exported proteins from the outer surface of the *Escherichia coli* plasma membrane. Journal of Biological Chemistry, 260, 15925–15931. 2999144

[mmi14461-bib-0029] De Buck, E. , Vranckx, L. , Meyen, E. , Maes, L. , Vandersmissen, L. , Anné, J. , & Lammertyn, E. (2007). The twin‐arginine translocation pathway is necessary for correct membrane insertion of the Rieske Fe/S protein in *Legionella pneumophila* . FEBS Letters, 581, 259–264. 17188684 10.1016/j.febslet.2006.12.022

[mmi14461-bib-0030] De Keersmaeker, S. , van Mellaert, L. , Lammertyn, E. , Vrancken, K. , Anné, J. , & Geukens, N. (2005). Functional analysis of TatA and TatB in *Streptomyces lividans* . Biochemical and Biophysical Research Communications, 335, 973–982. 10.1016/j.bbrc.2005.07.165 16111662

[mmi14461-bib-0031] de Leeuw, E. , Granjon, T. , Porcelli, I. , Alami, M. , Carr, S. B. , Müller, M. , … Berks, B. C. (2002). Oligomeric properties and signal peptide binding by *Escherichia coli* Tat protein transport complexes. Journal of Molecular Biology, 322, 1135–1146. 10.1016/S0022-2836(02)00820-3 12367533

[mmi14461-bib-0032] Delisa, M. P. , Samuelson, P. , Palmer, T. , & Georgiou, G. (2002). Genetic analysis of the twin arginine translocator secretion pathway in bacteria. Journal of Biological Chemistry, 277, 29825–29831. 10.1074/jbc.M201956200 12021272

[mmi14461-bib-0033] Delisa, M. P. , Tullman, D. , & Georgiou, G. (2003). Folding quality control in the export of proteins by the bacterial twin‐arginine translocation pathway. Proceedings of the National Academy of Sciences of the United States of America, 100, 6115–6120. 10.1073/pnas.0937838100 12721369 PMC156335

[mmi14461-bib-0034] Dilks, K. , Gimenez, M. I. , & Pohlschröder, M. (2005). Genetic and biochemical analysis of the twin‐arginine translocation pathway in halophilic archaea. Journal of Bacteriology, 187, 8104–8113. 10.1128/JB.187.23.8104-8113.2005 16291683 PMC1291277

[mmi14461-bib-0035] Fekkes, P. , De Wit, J. G. , van der Wolk, J. P. , Kimsey, H. H. , Kumamoto, C. A. , & Driessen, A. J. (1998). Preprotein transfer to the *Escherichia coli* translocon requires the co‐operative binding of SecB and the signal sequence to SecA. Molecular Microbiology, 29, 1179–1190. 9767586 10.1046/j.1365-2958.1998.00997.x

[mmi14461-bib-0036] Geller, B. , Zhu, H. Y. , Cheng, S. , Kuhn, A. , & Dalbey, R. E. (1993). Charged residues render pro‐OmpA potential dependent for initiation of membrane translocation. Journal of Biological Chemistry, 268, 9442–9447. 8486637

[mmi14461-bib-0037] Gérard, F. , & Cline, K. (2006). Efficient twin arginine translocation (Tat) pathway transport of a precursor protein covalently anchored to its initial cpTatC binding site. Journal of Biological Chemistry, 281, 6130–6135. 16407185 10.1074/jbc.M512733200

[mmi14461-bib-0038] Gérard, F. , & Cline, K. (2007). The thylakoid proton gradient promotes an advanced stage of signal peptide binding deep within the Tat pathway receptor complex. Journal of Biological Chemistry, 282, 5263–5272. 10.1074/jbc.M610337200 17172598

[mmi14461-bib-0039] Gohlke, U. , Pullan, L. , McDevitt, C. A. , Porcelli, I. , de Leeuw, E. , Palmer, T. , … Berks, B. C. (2005). The TatA component of the twin‐arginine protein transport system forms channel complexes of variable diameter. Proceedings of the National Academy of Sciences of the United States of America, 102, 10482–10486. 10.1073/pnas.0503558102 16027357 PMC1180781

[mmi14461-bib-0040] Goosens, V. J. , Monteferrante, C. G. , & van Dijl, J. M. (2014). Co‐factor insertion and disulfide bond requirements for twin‐arginine translocon‐dependent export of the *Bacillus subtilis* Rieske protein QcrA. Journal of Biological Chemistry, 289, 13124–13131. 24652282 10.1074/jbc.M113.529677 PMC4036324

[mmi14461-bib-0041] Habersetzer, J. , Moore, K. , Cherry, J. , Buchanan, G. , Stansfeld, P. J. , & Palmer, T . (2017). Substrate‐triggered position switching of TatA and TatB during Tat transport in *Escherichia coli* . Open Biology, 7, pii: 170091. 10.1098/rsob.170091 PMC5577447 28814647

[mmi14461-bib-0042] Halbig, D. , Wiegert, T. , Blaudeck, N. , Freudl, R. , & Sprenger, G. A. (1999). The efficient export of NADP‐containing glucose‐fructose oxidoreductase to the periplasm of *Zymomonas mobilis* depends both on an intact twin‐arginine motif in the signal peptide and on the generation of a structural export signal induced by cofactor binding. European Journal of Biochemistry, 263, 543–551. 10.1046/j.1432-1327.1999.00536.x 10406965

[mmi14461-bib-0043] Hamsanathan, S. , Anthonymuthu, T. S. , Bageshwar, U. K. , & Musser, S. M. (2017). A hinged signal peptide hairpin enables Tat‐dependent protein translocation. Biophysical Journal, 113, 2650–2668. 10.1016/j.bpj.2017.09.036 29262359 PMC5770558

[mmi14461-bib-0044] Hamsanathan, S. , & Musser, S. M. (2018). The Tat protein transport system: Intriguing questions and conundrums. FEMS Microbiology Letters, 365 10.1093/femsle/fny123 PMC5995166 29897510

[mmi14461-bib-0045] Hicks, M. G. , Guymer, D. , Buchanan, G. , Widdick, D. A. , Caldelari, I. , Berks, B. C. , & Palmer, T. (2006). Formation of functional Tat translocons from heterologous components. BMC Microbiology, 6, 64. 16854235 10.1186/1471-2180-6-64 PMC1550398

[mmi14461-bib-0046] Hinsley, A. P. , Stanley, N. R. , Palmer, T. , & Berks, B. C. (2001). A naturally occurring bacterial Tat signal peptide lacking one of the ‘invariant’ arginine residues of the consensus targeting motif. FEBS Letters, 497, 45–49. 10.1016/S0014-5793(01)02428-0 11376660

[mmi14461-bib-0047] Hopkins, A. , Buchanan, G. , & Pamer, T. (2014). Role of the twin arginine protein transport pathway in the assembly of the *Streptomyces coelicolor* cytochrome *bc* _1_ complex. Journal of Bacteriology, 196, 50–59. 10.1128/JB.00776-13 24142258 PMC3911139

[mmi14461-bib-0048] Hu, Y. , Zhao, E. , Li, H. , Xia, B. , & Jin, C. (2010). Solution NMR structure of the TatA component of the twin‐arginine protein transport system from gram‐positive bacterium *Bacillus subtilis* . Journal of the American Chemical Society, 132, 15942–15944. 20726548 10.1021/ja1053785

[mmi14461-bib-0049] Huang, Q. , Alcock, F. , Kneuper, H. , Deme, J. C. , Rollauer, S. E. , Lea, S. M. … Palmer, T . (2017). A signal sequence suppressor mutant that stabilizes an assembled state of the twin arginine translocon. Proceedings of the National Academy of Sciences of the United States of America, 114, E1958–E1967. 28223511 10.1073/pnas.1615056114 PMC5347605

[mmi14461-bib-0050] Huang, Q. , & Palmer, T. (2017). Signal peptide hydrophobicity modulates interaction with the twin‐arginine translocon. MBio, 8, pii: e00909–17. 10.1128/mBio.00909-17 PMC5539426 28765221

[mmi14461-bib-0051] Ize, B. , Stanley, N. R. , Buchanan, G. , & Palmer, T. (2003). Role of the *Escherichia coli* Tat pathway in outer membrane integrity. Molecular Microbiology, 48, 1183–1193. 10.1046/j.1365-2958.2003.03504.x 12787348

[mmi14461-bib-0052] Jongbloed, J. D. , Grieger, U. , Antelmann, H. , Hecker, M. , Nijland, R. , Bron, S. , & van Dijl, J. M. (2004). Two minimal Tat translocons in *Bacillus* . Molecular Microbiology, 54, 1319–1325. 15554971 10.1111/j.1365-2958.2004.04341.x

[mmi14461-bib-0053] Joshi, M. V. , Mann, S. G. , Antelmann, H. , Widdick, D. A. , Fyans, J. K. , Chandra, G. , … Palmer, T. (2010). The twin arginine protein transport pathway exports multiple virulence proteins in the plant pathogen *Streptomyces scabies* . Molecular Microbiology, 77, 252–271. 10.1111/j.1365-2958.2010.07206.x 20487278

[mmi14461-bib-0054] Keller, R. , De Keyzer, J. , Driessen, A. J. , & Palmer, T. (2012). Co‐operation between different targeting pathways during integration of a membrane protein. Journal of Cell Biology, 199, 303–315. 10.1083/jcb.201204149 23045547 PMC3471235

[mmi14461-bib-0055] Koch, S. , Fritsch, M. J. , Buchanan, G. , & Palmer, T. (2012). The *Escherichia coli* TatA and TatB proteins have an N‐out C‐in topology in intact cells. Journal of Biological Chemistry, 287, 14420–14431. 22399293 10.1074/jbc.M112.354555 PMC3340247

[mmi14461-bib-0056] Leake, M. C. , Greene, N. P. , Godun, R. M. , Granjon, T. , Buchanan, G. , Chen, S. … Berks, B. C . (2008). Variable stoichiometry of the TatA component of the twin‐arginine protein transport system observed by in vivo single‐molecule imaging. Proceedings of the National Academy of Sciences of the United States of America, 105, 15376–15381. 18832162 10.1073/pnas.0806338105 PMC2563114

[mmi14461-bib-0057] Li, P. , Beckwith, J. , & Inouye, H. (1988). Alteration of the amino terminus of the mature sequence of a periplasmic protein can severely affect protein export in *Escherichia coli* . Proceedings of the National Academy of Sciences of the United States of America, 85, 7685–7689. 10.1073/pnas.85.20.7685 3051001 PMC282257

[mmi14461-bib-0058] Lüke, I. , Handford, J. I. , Palmer, T. , & Sargent, F. (2009). Proteolytic processing *of Escherichia coli* twin‐arginine signal peptides by LepB. Archives of Microbiology, 191, 919–925. 19809807 10.1007/s00203-009-0516-5

[mmi14461-bib-0059] Lycklama a Nijeholt, J. A. , & Driessen, A. J . (2012). The bacterial Sec‐translocon: Structure and mechanism. Philosophical Transactions of the Royal Society of London. Series B, Biological Sciences, 367, 1016–1028. 22411975 10.1098/rstb.2011.0201 PMC3297432

[mmi14461-bib-0060] Ma, X. , & Cline, K. (2000). Precursors bind to specific sites on thylakoid membranes prior to transport on the delta pH protein translocation system. Journal of Biological Chemistry, 275, 10016–10022. 10744678 10.1074/jbc.275.14.10016

[mmi14461-bib-0061] Meloni, S. , Rey, L. , Sidler, S. , Imperial, J. , Ruiz‐Argueso, T. , & Palacios, J. M. (2003). The twin‐arginine translocation (Tat) system is essential for *Rhizobium*‐legume symbiosis. Molecular Microbiology, 48, 1195–1207. 10.1046/j.1365-2958.2003.03510.x 12787349

[mmi14461-bib-0062] Molik, S. , Karnauchov, I. , Weidlich, C. , Herrmann, R. G. , & Klösgen, R. B. (2001). The Rieske Fe/S protein of the cytochrome *b* _6_/*f* complex in chloroplasts: Missing link in the evolution of protein transport pathways in chloroplasts? Journal of Biological Chemistry, 276, 42761–42766. 11526115 10.1074/jbc.M106690200

[mmi14461-bib-0063] Mori, H. , & Cline, K. (2002). A twin arginine signal peptide and the pH gradient trigger reversible assembly of the thylakoid [Delta]pH/Tat translocon. Journal of Cell Biology, 157, 205–210. 11956224 10.1083/jcb.200202048 PMC2199252

[mmi14461-bib-0064] Musser, S. M. , & Theg, S. M. (2000). Characterization of the early steps of OE17 precursor transport by the thylakoid DeltapH/Tat machinery. European Journal of Biochemistry, 267, 2588–2598. 10785379 10.1046/j.1432-1327.2000.01269.x

[mmi14461-bib-0065] Niebisch, A. , & Bott, M. (2001). Molecular analysis of the cytochrome *bc* _1_‐*aa* _3_ branch of the *Corynebacterium glutamicum* respiratory chain containing an unusual diheme cytochrome *c* _1_ . Archives of Microbiology, 175, 282–294. 10.1007/s002030100262 11382224

[mmi14461-bib-0066] Nunez, P. A. , Soria, M. , & Farber, M. D. (2012). The twin‐arginine translocation pathway in alpha‐proteobacteria is functionally preserved irrespective of genomic and regulatory divergence. PLoS One, 7, e33605. 22438962 10.1371/journal.pone.0033605 PMC3305326

[mmi14461-bib-0067] Oates, J. , Barrett, C. M. , Barnett, J. P. , Byrne, K. G. , Bolhuis, A. , & Robinson, C. (2005). The *Escherichia coli* twin‐arginine translocation apparatus incorporates a distinct form of TatABC complex, spectrum of modular TatA complexes and minor TatAB complex. Journal of Molecular Biology, 346, 295–305. 10.1016/j.jmb.2004.11.047 15663945

[mmi14461-bib-0068] Palmer, T. , & Berks, B. C. (2012). The twin‐arginine translocation (Tat) protein export pathway. Nature Reviews Microbiology, 10, 483–496. 10.1038/nrmicro2814 22683878

[mmi14461-bib-0069] Palmer, T. , Sargent, F. , & Berks, B. C. (2010). The Tat protein export pathway. EcoSal Plus, 4 10.1128/ecosalplus.4.3.2 26443788

[mmi14461-bib-0070] Panahandeh, S. , Maurer, C. , Moser, M. , Delisa, M. P. , & Müller, M. (2008). Following the path of a twin‐arginine precursor along the TatABC translocon of *Escherichia coli* . Journal of Biological Chemistry, 283, 33267–33275. 18836181 10.1074/jbc.M804225200 PMC2662257

[mmi14461-bib-0071] Petru, M. , Wideman, J. , Moore, K. , Alcock, F. , Palmer, T. , & Dolezal, P. (2018). Evolution of mitochondrial TAT translocons illustrates the loss of bacterial protein transport machines in mitochondria. BMC Biology, 16, 141. 30466434 10.1186/s12915-018-0607-3 PMC6251230

[mmi14461-bib-0072] Pett, W. , & Lavrov, D. V. (2013). The twin‐arginine subunit C in *Oscarella*: Origin, evolution, and potential functional significance. Integrative & Comparative Biology, 53, 495–502. 10.1093/icb/ict079 23864529

[mmi14461-bib-0073] Ramasamy, S. , Abrol, R. , Suloway, C. J. , & Clemons, W. M . (2013). The glove‐like structure of the conserved membrane protein TatC provides insight into signal sequence recognition in twin‐arginine translocation. Structure, 21, 777–788. 10.1016/j.str.2013.03.004 23583035 PMC3653977

[mmi14461-bib-0074] Rapoport, T. A. , Li, L. , & Park, E. (2017). Structural and mechanistic insights into protein translocation. Annual Review of Cell and Developmental Biology, 33, 369–390. 10.1146/annurev-cellbio-100616-060439 28564553

[mmi14461-bib-0075] Richter, S. , & Büser, T. (2005). Targeting of unfolded PhoA to the TAT translocon of *Escherichia coli* . Journal of Biological Chemistry, 280, 42723–42730. 16263723 10.1074/jbc.M509570200

[mmi14461-bib-0076] Rodriguez, F. , Rouse, S. L. , Tait, C. E. , Harmer, J. , De Riso, A. , Timmel, C. R. , … Schnell, J. R. (2013). Structural model for the protein‐translocating element of the twin‐arginine transport system. Proceedings of the National Academy of Sciences of the United States of America, 110, E1092–E1101. 10.1073/pnas.1219486110 23471988 PMC3607022

[mmi14461-bib-0077] Rollauer, S. E. , Tarry, M. J. , Graham, J. E. , Jääskeläinen, M. , Jäger, F. , Johnson, S. , … Lea, S. M. (2012). Structure of the TatC core of the twin‐arginine protein transport system. Nature, 492, 210–214. 10.1038/nature11683 23201679 PMC3573685

[mmi14461-bib-0078] Rose, P. , Fröbel, J. , Graumann, P. L. , & Müller, M. (2013). Substrate‐dependent assembly of the Tat translocon as observed in live *Escherichia coli* cells. PLoS ONE, 8, e69488. 23936332 10.1371/journal.pone.0069488 PMC3732296

[mmi14461-bib-0079] Santini, C. L. , Ize, B. , Chanal, A. , Müller, M. , Giordano, G. , & Wu, L. F. (1998). A novel sec‐independent periplasmic protein translocation pathway in *Escherichia coli* . EMBO Journal, 17, 101–112. 10.1093/emboj/17.1.101 9427745 PMC1170362

[mmi14461-bib-0080] Sargent, F. , Bogsch, E. G. , Stanley, N. R. , Wexler, M. , Robinson, C. , Berks, B. C. , & Palmer, T. (1998). Overlapping functions of components of a bacterial Sec‐independent protein export pathway. EMBO Journal, 17, 3640–3650. 10.1093/emboj/17.13.3640 9649434 PMC1170700

[mmi14461-bib-0081] Sargent, F. , Stanley, N. R. , Berks, B. C. , & Palmer, T. (1999). Sec‐independent protein translocation in *Escherichia coli*. A distinct and pivotal role for the TatB protein. Journal of Biological Chemistry, 274, 36073–36082. 10593889 10.1074/jbc.274.51.36073

[mmi14461-bib-0082] Sasaki, S. , Matsuyama, S. , & Mizushima, S. (1990). In vitro kinetic analysis of the role of the positive charge at the amino‐terminal region of signal peptides in translocation of secretory protein across the cytoplasmic membrane in *Escherichia coli* . Journal of Biological Chemistry, 265, 4358–4363. 2106519

[mmi14461-bib-0083] Schierle, C. F. , Berkmen, M. , Huber, D. , Kumamoto, C. , Boyd, D. , & Beckwith, J. (2003). The DsbA signal sequence directs efficient, cotranslational export of passenger proteins to the *Escherichia coli* periplasm via the signal recognition particle pathway. Journal of Bacteriology, 185, 5706–5713. 13129941 10.1128/JB.185.19.5706-5713.2003 PMC193964

[mmi14461-bib-0084] Settles, A. M. , Yonetani, A. , Baron, A. , Bush, D. R. , Cline, K. , & Martienssen, R. (1997). Sec‐independent protein translocation by the maize Hcf106 protein. Science, 278, 1467–1470. 10.1126/science.278.5342.1467 9367960

[mmi14461-bib-0085] Shanmugham, A. , Wong Fong Sang, H. W. , Bollen, Y. J. M. , & Lill, H. (2006). Membrane binding of twin arginine preproteins as an early step in translocation. Biochemistry, 45, 2243–2249. 10.1021/bi052188a 16475812

[mmi14461-bib-0086] Stanley, N. R. , Palmer, T. , & Berks, B. C. (2000). The twin arginine consensus motif of Tat signal peptides is involved in Sec‐independent protein targeting in *Escherichia coli* . Journal of Biological Chemistry, 275, 11591–11596. 10766774 10.1074/jbc.275.16.11591

[mmi14461-bib-0087] Stull, F. , Betton, J. M. , & Bardwell, J. C. A. (2018). Periplasmic chaperones and prolyl isomerases. EcoSal Plus, 8 10.1128/ecosalplus.ESP-0005-2018 PMC11575675 29988001

[mmi14461-bib-0088] Tarry, M. J. , Schafer, E. , Chen, S. , Buchanan, G. , Greene, N. P. , Lea, S. M. , … Berks, B. C. (2009). Structural analysis of substrate binding by the TatBC component of the twin‐arginine protein transport system. Proceedings of the National Academy of Sciences of the United States of America, 106, 13284–13289. 10.1073/pnas.0901566106 19666509 PMC2718047

[mmi14461-bib-0089] Tooke, F. J. , Babot, M. , Chandra, G. , Buchanan, G. , & Palmer, T . (2017). A unifying mechanism for the biogenesis of membrane proteins co‐operatively integrated by the Sec and Tat pathways. Elife, 6, pii: e26577. 10.7554/eLife.26577 PMC5449189 28513434

[mmi14461-bib-0090] Tsirigotaki, A. , de Geyter, J. , Sostaric, N. , Economou, A. , & Karamanou, S. (2017). Protein export through the bacterial Sec pathway. Nature Reviews Microbiology, 15, 21–36. 10.1038/nrmicro.2016.161 27890920

[mmi14461-bib-0091] Tullman‐Ercek, D. , Delisa, M. P. , Kawarasaki, Y. , Iranpour, P. , Ribnicky, B. , Palmer, T. , & Georgiou, G. (2007). Export pathway selectivity of *Escherichia coli* twin arginine translocation signal peptides. Journal of Biological Chemistry, 282, 8309–8316. 17218314 10.1074/jbc.M610507200 PMC2730154

[mmi14461-bib-0092] Ulfig, A. , & Freudl, R. (2018). The early mature part of bacterial twin‐arginine translocation (Tat) precursor proteins contributes to TatBC receptor binding. Journal of Biological Chemistry, 293, 7281–7299. 10.1074/jbc.RA118.002576 29593092 PMC5949997

[mmi14461-bib-0093] Ulfig, A. , Fröbel, J. , Lausberg, F. , Blümmel, A. S. , Heide, A. K. , Müller, M. , & Freudl, R. (2017). The h‐region of twin arginine signal peptides supports productive binding of bacterial Tat precursor proteins to the TatBC receptor complex. Journal of Biological Chemistry, 292, 10865–10882. 10.1074/jbc.M117.788950 28515319 PMC5491773

[mmi14461-bib-0094] Van Stelten, J. , Silva, F. , Belin, D. , & Silhavy, T. J. (2009). Effects of antibiotics and a proto‐oncogene homolog on destruction of protein translocator SecY. Science, 325, 753–756. 10.1126/science.1172221 19661432 PMC2832214

[mmi14461-bib-0095] Weiner, J. H. , Bilous, P. T. , Shaw, G. M. , Lubitz, S. P. , Frost, L. , Thomas, G. H. , … Turner, R. J. (1998). A novel and ubiquitous system for membrane targeting and secretion of cofactor‐containing proteins. Cell, 93, 93–101. 10.1016/S0092-8674(00)81149-6 9546395

[mmi14461-bib-0096] Widdick, D. A. , Dilks, K. , Chandra, G. , Bottrill, A. , Naldrett, M. , Pohlschröder, M. , & Palmer, T. (2006). The twin‐arginine translocation pathway is a major route of protein export in *Streptomyces coelicolor* . Proceedings of the National Academy of Sciences of the United States of America, 103, 17927–17932. 10.1073/pnas.0607025103 17093047 PMC1693849

[mmi14461-bib-0097] Willemse, J. , Ruban‐Osmialowska, B. , Widdick, D. , Celler, K. , Hutchings, M. I. , van Wezel, G. P. , & Palmer, T. (2012). Dynamic localization of Tat protein transport machinery components in *Streptomyces coelicolor* . Journal of Bacteriology, 194, 6272–6281. 10.1128/JB.01425-12 23002216 PMC3486365

[mmi14461-bib-0098] Yahr, T. L. , & Wickner, W. T. (2001). Functional reconstitution of bacterial Tat translocation in vitro. EMBO Journal, 20, 2472–2479. 11350936 10.1093/emboj/20.10.2472 PMC125449

[mmi14461-bib-0099] Zhang, Y. , Wang, L. , Hu, Y. , & Jin, C. (2014). Solution structure of the TatB component of the twin‐arginine translocation system. Biochimica et Biophysica Acta, 1838, 1881–1888. 24699374 10.1016/j.bbamem.2014.03.015

[mmi14461-bib-0100] Zoufaly, S. , Fröbel, J. , Rose, P. , Flecken, T. , Maurer, C. , Moser, M. , & Müller, M. (2012). Mapping precursor‐binding site on TatC subunit of twin arginine‐specific protein translocon by site‐specific photo cross‐linking. Journal of Biological Chemistry, 287, 13430–13441. 22362773 10.1074/jbc.M112.343798 PMC3339946

